# (*Z*)-*N*-[3-(Phenyl­sulfon­yl)thia­zolidin-2-yl­idene]cyanamide

**DOI:** 10.1107/S1600536810041371

**Published:** 2010-10-20

**Authors:** Jian Hou

**Affiliations:** a Science and Technology of Marine Corrosion and Protection Laboratory, Luoyang Ship Material Research Institute, Qingdao 266101, People’s Republic of China

## Abstract

In the title compound, C_10_H_9_N_3_O_2_S_2_, the dihedral angle between the benzene and thia­zolidine rings is 79.8 (2)°. Inter­molecular C—H⋯N and C—H⋯O inter­actions help to stabilize the crystal structure.

## Related literature

For related structures, see: Wang *et al.* (2008 ▶); Liu & Li (2009 ▶); Xie & Li (2010 ▶). For details of the corrosion inhibition activity of thia­zolidine-containing compounds, see: Trabanelli (1991 ▶); Jardy *et al.* (1992 ▶); Sarawy *et al.* (2008 ▶); Vastag *et al.* (2001 ▶). For bond-length data, see: Allen *et al.* (1987 ▶).
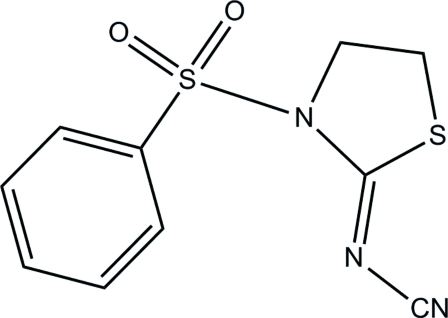


## Experimental

### 

#### Crystal data

C_10_H_9_N_3_O_2_S_2_
*M* *_r_* = 267.32
Tetragonal, 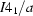

*a* = 15.186 (2) Å
*c* = 19.858 (4) Å
*V* = 4579.7 (13) Å^3^
*Z* = 16
Mo *K*α radiation
μ = 0.46 mm^−1^
*T* = 173 K
0.60 × 0.50 × 0.40 mm

#### Data collection

Rigaku Mercury CCD/AFC diffractometer
Absorption correction: multi-scan (*CrystalClear*; Rigaku, 2007 ▶) *T*_min_ = 0.771, *T*_max_ = 0.838
8388 measured reflections
2020 independent reflections
1968 reflections with *I* > 2σ(*I*)
*R*_int_ = 0.043

#### Refinement

*R*[*F* ^2^ > 2σ(*F* ^2^)] = 0.048
*wR*(*F* ^2^) = 0.143
*S* = 1.26
2020 reflections
154 parameters
H-atom parameters constrained
Δρ_max_ = 0.27 e Å^−3^
Δρ_min_ = −0.38 e Å^−3^

### 

Data collection: *CrystalClear* (Rigaku, 2007 ▶); cell refinement: *CrystalClear*; data reduction: *CrystalClear*; program(s) used to solve structure: *SHELXS97* (Sheldrick, 2008 ▶); program(s) used to refine structure: *SHELXL97* (Sheldrick, 2008 ▶); molecular graphics: *SHELXTL* (Sheldrick, 2008 ▶) (Sheldrick, 2008 ▶); software used to prepare material for publication: *SHELXTL*.

## Supplementary Material

Crystal structure: contains datablocks I, global. DOI: 10.1107/S1600536810041371/hg2727sup1.cif

Structure factors: contains datablocks I. DOI: 10.1107/S1600536810041371/hg2727Isup2.hkl

Additional supplementary materials:  crystallographic information; 3D view; checkCIF report

## Figures and Tables

**Table 1:** Hydrogen-bond geometry (Å, °)

*D*—H⋯*A*	*D*—H	H⋯*A*	*D*⋯*A*	*D*—H⋯*A*
C2—H2*C*⋯N3^i^	0.93	2.60	3.349 (4)	138
C4—H4*A*⋯O1^ii^	0.93	2.58	3.290 (4)	133
C7—H7*B*⋯O2^iii^	0.97	2.60	3.169 (4)	118
C7—H7*A*⋯O2^iv^	0.97	2.55	3.506 (4)	168
C8—H8*A*⋯O1^v^	0.97	2.56	3.283 (4)	131
C8—H8*B*⋯N3^vi^	0.97	2.58	3.299 (5)	131

Symmetry codes: (i) 
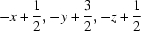
; (ii) 
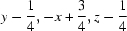
; (iii) 
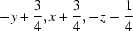
; (iv) 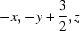; (v) 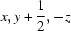; (vi) 
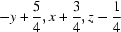
.

**Fig. 1.**
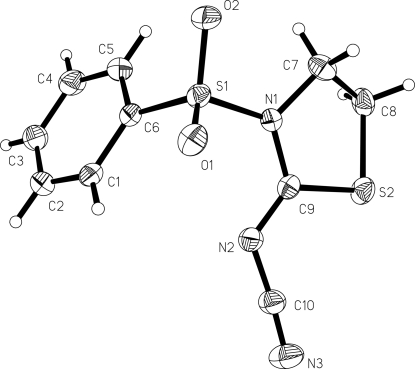
The molecular structure of (I), with atom labels and 40% probability displacement ellipsoids for non-H atoms.

## supplementary crystallographic information

### Comment

Thiazolidine is an important kind of group in organic chemistry. The molecular structure of thiazole contains N and S atoms, which are easily able to bridge with other molecules or metals (Trabanelli, 1991; Jardy *et al.*, 1992). And many researchers have been focused on the corrosion inhibition performance of the thiazole. Sarawy (Sarawy *et al.*, 2008) used the weight loss and electrochemical polarization methods studied some thiazole derivatives as corrosion inhibitors for carbon steel in acidic medium. Vastag (Vastag *et al.*, 2001) investigated the inhibition characteristics of some thiazole derivatives against copper corrosion in acidic sulfate containing media. In order to search for new thiazole compounds with higher corrosion inhibition, we synthesized the (*Z*)—*N*-(3-(phenylsulfonyl) thiazolidin-2-ylidene)cyanamide and describe its structure here.

In title compound, all bond lengths in the molecular are normal (Allen *et al.*, 1987) and in a good agreement with those reported previously (Wang *et al.*, 2008; Liu & Li, 2009; Xie & Li, 2010). The dihedral angle between benzene (C1—C6) and thiazolidine (C7—C9/N1/S2) rings is 79.8 (2) °. The intermolecular C—H···N and C—H···O hydrogen bonds stabilize the structure.

### Experimental

A mixture of *N*-cyanoiminothiazolidine 10 mmol (1.27 g), benzenesulfonyl chloride (1.77 g, 10 mmol) and (1.01 g, 10 mmol) triethylamine is refluxed in absolute acetone (25 ml) for 3 h. On cooling, the product crystallizes and is filtered, and recrystallized from absolute EtOH, yield 2.38 g (89.3%). Single crystals suitable for X-ray measurements were obtained by recrystallization from acetonitrile at room temperature.

### Refinement

H atoms were positioned geometrically and refined using a riding model, with C—H = 0.93 or 0.97 Å and with *U*_iso_(H) = 1.2 times *U*_eq_(C).

### Crystal data

**Table:**

C_10_H_9_N_3_O_2_S_2_	*D*_x_ = 1.551 Mg m^−^^3^
*M**_r_* = 267.32	Mo *K*α radiation, λ = 0.71073 Å
Tetragonal, *I*4_1_/*a*	Cell parameters from 7269 reflections
Hall symbol: -I 4ad	θ = 1.7–27.5°
*a* = 15.186 (2) Å	µ = 0.46 mm^−^^1^
*c* = 19.858 (4) Å	*T* = 173 K
*V* = 4579.7 (13) Å^3^	Block, colorless
*Z* = 16	0.60 × 0.50 × 0.40 mm
*F*(000) = 2208	

### Data collection

**Table:**

Rigaku Mercury CCD/AFC diffractometer	2020 independent reflections
Radiation source: Sealed Tube	1968 reflections with *I* > 2σ(*I*)
Graphite Monochromator	*R*_int_ = 0.043
φ and ω scans	θ_max_ = 25.0°, θ_min_ = 1.7°
Absorption correction: multi-scan (*CrystalClear*; Rigaku, 2007)	*h* = −18→16
*T*_min_ = 0.771, *T*_max_ = 0.838	*k* = −18→17
8388 measured reflections	*l* = −23→16

### Refinement

**Table:**

Refinement on *F*^2^	Primary atom site location: structure-invariant direct methods
Least-squares matrix: full	Secondary atom site location: difference Fourier map
*R*[*F*^2^ > 2σ(*F*^2^)] = 0.048	Hydrogen site location: inferred from neighbouring sites
*wR*(*F*^2^) = 0.143	H-atom parameters constrained
*S* = 1.25	*w* = 1/[σ^2^(*F*_o_^2^) + (0.0646*P*)^2^ + 5.9285*P*] where *P* = (*F*_o_^2^ + 2*F*_c_^2^)/3
2020 reflections	(Δ/σ)_max_ < 0.001
154 parameters	Δρ_max_ = 0.27 e Å^−^^3^
0 restraints	Δρ_min_ = −0.38 e Å^−^^3^

### Special details

**Table:**

Geometry. All e.s.d.'s (except the e.s.d. in the dihedral angle between two l.s. planes) are estimated using the full covariance matrix. The cell e.s.d.'s are taken into account individually in the estimation of e.s.d.'s in distances, angles and torsion angles; correlations between e.s.d.'s in cell parameters are only used when they are defined by crystal symmetry. An approximate (isotropic) treatment of cell e.s.d.'s is used for estimating e.s.d.'s involving l.s. planes.
Refinement. Refinement of *F*^2^ against ALL reflections. The weighted *R*-factor *wR* and goodness of fit *S* are based on *F*^2^, conventional *R*-factors *R* are based on *F*, with *F* set to zero for negative *F*^2^. The threshold expression of *F*^2^ > σ(*F*^2^) is used only for calculating *R*-factors(gt) *etc*. and is not relevant to the choice of reflections for refinement. *R*-factors based on *F*^2^ are statistically about twice as large as those based on *F*, and *R*- factors based on ALL data will be even larger.

### Fractional atomic coordinates and isotropic or equivalent isotropic displacement parameters (Å^2^)

**Table:**

	*x*	*y*	*z*	*U*_iso_*/*U*_eq_	
S1	0.13963 (5)	0.70489 (5)	0.01490 (4)	0.0282 (3)	
S2	0.12354 (6)	0.97377 (5)	0.06596 (4)	0.0357 (3)	
O1	0.08728 (14)	0.66766 (13)	0.06712 (11)	0.0350 (5)	
O2	0.11758 (15)	0.68682 (15)	−0.05369 (11)	0.0383 (6)	
N1	0.13300 (16)	0.81530 (16)	0.01935 (11)	0.0274 (6)	
N2	0.15682 (17)	0.82265 (16)	0.13475 (12)	0.0311 (6)	
N3	0.1687 (2)	0.9108 (2)	0.23973 (14)	0.0486 (8)	
C1	0.2804 (2)	0.65169 (19)	0.09004 (15)	0.0314 (7)	
H1B	0.2417	0.6458	0.1260	0.038*	
C2	0.3685 (2)	0.6318 (2)	0.09725 (16)	0.0381 (8)	
H2C	0.3895	0.6117	0.1385	0.046*	
C3	0.4257 (2)	0.6414 (2)	0.04396 (17)	0.0411 (8)	
H3A	0.4851	0.6283	0.0498	0.049*	
C4	0.3960 (2)	0.6701 (2)	−0.01812 (17)	0.0413 (8)	
H4A	0.4350	0.6760	−0.0539	0.050*	
C5	0.3083 (2)	0.6900 (2)	−0.02651 (15)	0.0339 (7)	
H5A	0.2875	0.7095	−0.0680	0.041*	
C6	0.25123 (19)	0.68082 (18)	0.02760 (14)	0.0276 (6)	
C7	0.1061 (2)	0.8673 (2)	−0.04017 (16)	0.0356 (7)	
H7A	0.0436	0.8602	−0.0485	0.043*	
H7B	0.1381	0.8479	−0.0798	0.043*	
C8	0.1273 (2)	0.9626 (2)	−0.02480 (16)	0.0364 (8)	
H8A	0.0844	1.0012	−0.0458	0.044*	
H8B	0.1853	0.9776	−0.0416	0.044*	
C9	0.13968 (18)	0.86106 (19)	0.07808 (15)	0.0275 (6)	
C10	0.1631 (2)	0.8732 (2)	0.18980 (16)	0.0348 (7)	

### Atomic displacement parameters (Å^2^)

**Table:**

	*U*^11^	*U*^22^	*U*^33^	*U*^12^	*U*^13^	*U*^23^
S1	0.0299 (4)	0.0242 (4)	0.0305 (4)	0.0001 (3)	−0.0031 (3)	−0.0052 (3)
S2	0.0449 (5)	0.0228 (4)	0.0395 (5)	0.0024 (3)	0.0046 (3)	0.0008 (3)
O1	0.0346 (12)	0.0276 (11)	0.0428 (13)	−0.0057 (9)	0.0050 (10)	−0.0013 (9)
O2	0.0412 (13)	0.0409 (13)	0.0328 (12)	0.0012 (10)	−0.0095 (10)	−0.0112 (10)
N1	0.0305 (13)	0.0254 (13)	0.0262 (13)	0.0041 (10)	−0.0030 (10)	−0.0001 (10)
N2	0.0413 (15)	0.0238 (12)	0.0281 (13)	0.0008 (11)	−0.0007 (11)	−0.0005 (10)
N3	0.067 (2)	0.0454 (17)	0.0333 (16)	−0.0070 (15)	0.0045 (14)	−0.0071 (14)
C1	0.0399 (17)	0.0268 (15)	0.0277 (15)	0.0030 (12)	0.0015 (13)	−0.0041 (12)
C2	0.0449 (19)	0.0376 (18)	0.0318 (17)	0.0078 (14)	−0.0072 (14)	−0.0011 (14)
C3	0.0357 (17)	0.048 (2)	0.0398 (18)	0.0104 (15)	−0.0061 (14)	−0.0054 (15)
C4	0.0365 (18)	0.052 (2)	0.0357 (18)	0.0059 (15)	0.0070 (14)	−0.0040 (15)
C5	0.0373 (17)	0.0362 (17)	0.0283 (15)	0.0017 (13)	0.0008 (13)	−0.0003 (13)
C6	0.0310 (15)	0.0237 (14)	0.0279 (15)	0.0023 (12)	−0.0017 (12)	−0.0035 (12)
C7	0.0359 (17)	0.0409 (18)	0.0301 (16)	−0.0042 (14)	−0.0023 (13)	0.0076 (14)
C8	0.0397 (18)	0.0315 (17)	0.0379 (18)	0.0055 (14)	0.0016 (14)	0.0107 (13)
C9	0.0253 (14)	0.0265 (15)	0.0308 (15)	0.0003 (11)	0.0023 (12)	−0.0023 (12)
C10	0.0464 (19)	0.0285 (16)	0.0295 (17)	−0.0021 (14)	0.0017 (14)	0.0012 (13)

### Geometric parameters (Å, °)

**Table:**

S1—O1	1.424 (2)	C2—C3	1.376 (5)
S1—O2	1.429 (2)	C2—H2C	0.9300
S1—N1	1.682 (2)	C3—C4	1.384 (5)
S1—C6	1.752 (3)	C3—H3A	0.9300
S2—C9	1.746 (3)	C4—C5	1.376 (4)
S2—C8	1.811 (3)	C4—H4A	0.9300
N1—C9	1.361 (4)	C5—C6	1.387 (4)
N1—C7	1.479 (4)	C5—H5A	0.9300
N2—C9	1.294 (4)	C7—C8	1.513 (4)
N2—C10	1.339 (4)	C7—H7A	0.9700
N3—C10	1.148 (4)	C7—H7B	0.9700
C1—C2	1.379 (4)	C8—H8A	0.9700
C1—C6	1.389 (4)	C8—H8B	0.9700
C1—H1B	0.9300		
			
O1—S1—O2	119.15 (14)	C4—C5—C6	119.3 (3)
O1—S1—N1	108.91 (12)	C4—C5—H5A	120.4
O2—S1—N1	103.14 (12)	C6—C5—H5A	120.4
O1—S1—C6	110.64 (14)	C5—C6—C1	121.6 (3)
O2—S1—C6	108.89 (14)	C5—C6—S1	118.1 (2)
N1—S1—C6	104.97 (13)	C1—C6—S1	120.3 (2)
C9—S2—C8	92.33 (14)	N1—C7—C8	106.9 (3)
C9—N1—C7	115.7 (2)	N1—C7—H7A	110.3
C9—N1—S1	123.3 (2)	C8—C7—H7A	110.3
C7—N1—S1	120.4 (2)	N1—C7—H7B	110.3
C9—N2—C10	117.8 (3)	C8—C7—H7B	110.3
C2—C1—C6	118.2 (3)	H7A—C7—H7B	108.6
C2—C1—H1B	120.9	C7—C8—S2	106.5 (2)
C6—C1—H1B	120.9	C7—C8—H8A	110.4
C3—C2—C1	120.6 (3)	S2—C8—H8A	110.4
C3—C2—H2C	119.7	C7—C8—H8B	110.4
C1—C2—H2C	119.7	S2—C8—H8B	110.4
C2—C3—C4	120.8 (3)	H8A—C8—H8B	108.6
C2—C3—H3A	119.6	N2—C9—N1	122.0 (3)
C4—C3—H3A	119.6	N2—C9—S2	126.2 (2)
C5—C4—C3	119.5 (3)	N1—C9—S2	111.8 (2)
C5—C4—H4A	120.2	N3—C10—N2	174.9 (3)
C3—C4—H4A	120.2		
			
O1—S1—N1—C9	45.1 (3)	O1—S1—C6—C1	−16.3 (3)
O2—S1—N1—C9	172.6 (2)	O2—S1—C6—C1	−149.1 (2)
C6—S1—N1—C9	−73.4 (3)	N1—S1—C6—C1	101.0 (2)
O1—S1—N1—C7	−125.7 (2)	C9—N1—C7—C8	21.5 (4)
O2—S1—N1—C7	1.8 (3)	S1—N1—C7—C8	−167.0 (2)
C6—S1—N1—C7	115.8 (2)	N1—C7—C8—S2	−26.8 (3)
C6—C1—C2—C3	0.5 (5)	C9—S2—C8—C7	21.5 (2)
C1—C2—C3—C4	−0.8 (5)	C10—N2—C9—N1	179.3 (3)
C2—C3—C4—C5	0.5 (5)	C10—N2—C9—S2	−0.1 (4)
C3—C4—C5—C6	0.0 (5)	C7—N1—C9—N2	175.3 (3)
C4—C5—C6—C1	−0.2 (5)	S1—N1—C9—N2	4.1 (4)
C4—C5—C6—S1	−178.9 (2)	C7—N1—C9—S2	−5.3 (3)
C2—C1—C6—C5	−0.1 (4)	S1—N1—C9—S2	−176.48 (15)
C2—C1—C6—S1	178.6 (2)	C8—S2—C9—N2	169.3 (3)
O1—S1—C6—C5	162.4 (2)	C8—S2—C9—N1	−10.1 (2)
O2—S1—C6—C5	29.6 (3)	C9—N2—C10—N3	171 (4)
N1—S1—C6—C5	−80.2 (3)		

### Hydrogen-bond geometry (Å, °)

**Table:**

*D*—H···*A*	*D*—H	H···*A*	*D*···*A*	*D*—H···*A*
C2—H2C···N3^i^	0.93	2.60	3.349 (4)	138
C4—H4A···O1^ii^	0.93	2.58	3.290 (4)	133
C7—H7B···O2^iii^	0.97	2.60	3.169 (4)	118
C7—H7A···O2^iv^	0.97	2.55	3.506 (4)	168
C8—H8A···O1^v^	0.97	2.56	3.283 (4)	131
C8—H8B···N3^vi^	0.97	2.58	3.299 (5)	131

Symmetry codes: (i) −*x*+1/2, −*y*+3/2, −*z*+1/2; (ii) *y*−1/4, −*x*+3/4, *z*−1/4; (iii) −*y*+3/4, *x*+3/4, −*z*−1/4; (iv) −*x*, −*y*+3/2, *z*; (v) *x*, *y*+1/2, −*z*; (vi) −*y*+5/4, *x*+3/4, *z*−1/4.
